# The relationship between the laboratory diagnosis of Lyme neuroborreliosis and climate factors in Kalmar County Sweden — an overview between 2008 and 2019

**DOI:** 10.1007/s10096-021-04374-4

**Published:** 2021-11-09

**Authors:** Kimberly Keith, Kristofer Årestedt, Ivar Tjernberg

**Affiliations:** 1 Medical Programme, Faculty of Medicine and Health Sciences, Linköping University, Linköping, Sweden; 2 Faculty of Health and Life Sciences, Linnaeus University, Kalmar, Sweden; 3 The Research Section, Region Kalmar County, Kalmar, Sweden; 4 Department of Clinical Chemistry and Transfusion Medicine, Region Kalmar County, Kalmar and Department of Biomedical and Clinical Sciences, Linköping University, Linköping, Sweden

**Keywords:** Lyme neuroborreliosis, Lyme borreliosis, Epidemiology, Incidence, Climate

## Abstract

The purpose of this study was to describe the epidemiology of Lyme neuroborreliosis (LNB) in Kalmar County, in southern Sweden, between 2008 and 2019, and to analyse the relationship between the LNB incidence and climate factors. Data containing cerebrospinal fluid (CSF) cell counts and borrelia CSF/serum antibody index results was received from the departments of clinical chemistry and microbiology at Kalmar County hospital. For this study, we defined LNB as a case with a positive borrelia antibody CSF/serum index and CSF leukocytes > 5 × 10^6^/L. Climate data including mean temperature, humidity and precipitation covering Kalmar County was collected from the Swedish Meteorological and Hydrological Institute. A total of 5051 paired serum-CSF samples from 4835 patients were investigated of which 251 laboratory LNB cases were found. The average annual LNB incidence in Kalmar County 2008–2019 was 8.8 cases per 100,000 inhabitants. Positive relationships were observed between mean temperature and LNB incidence (p < 0.001) as well as precipitation and LNB incidence (p = 0.003), both with a one calendar month delay. The results suggest an association between climate factors such as mean temperature and precipitation and LNB incidence, presumably through increased/decreased human-tick interactions. This calls for increased awareness of LNB in both the short perspective after periods of warmth and heavy precipitation as well as in a longer perspective in relation to possible climate change. Further studies with larger study groups, covering other geographical areas and over longer periods of time are needed to confirm these findings.

**Supplementary Information:**

The online version contains supplementary material available at 10.1007/s10096-021-04374-4.

## Introduction

European Lyme borreliosis (LB) is caused by spirochetes of the *Borrelia burgdorferi* sensu lato species complex, which is carried by the tick *Ixodes (I.) ricinus*. LB manifests in varying ways, the most common being erythema migrans (EM), a skin manifestation, and the second most common manifestation being Lyme neuroborreliosis (LNB) [1, 2]. Diagnosis of LNB is based on clinical symptoms as well as analysis of cerebrospinal fluid (CSF). According to recommendations by the EFNS guidelines, definite LNB can be diagnosed when the following three criteria are fulfilled; (I) neurological symptoms of LNB, with no other cause; (II) CSF pleocytosis; (III) intrathecal borrelia-specific antibodies in CSF. To prove that there are intrathecally produced antibodies, a CSF/serum index is used [1, 3].

LB is highly endemic in southern Sweden, including Kalmar County, the area for the current study. In addition, recent studies from Sweden and other European countries have shown that the LNB incidence has increased in the late 1990s and early 2000s [2, 4–7]. Incidence of LB is affected by several different parameters, including the frequency and abundance of borrelia positive ticks, as well as human exposure and behaviour. It has also been shown that climate factors may influence LB incidence. A Swedish study reported that the incidence of EM correlates positively with milder winters and warmer summers, as well as relative humidity levels above 86%. The same study also showed a negative correlation with mean monthly precipitation [8].

The increase in LB is thought to be due to an increase in the distribution and density of *I. ricinus*, which is partially affected by climate factors [9–13]. The number of infected ticks also affects the number of LB cases, in southern Sweden some 15–17% of ticks are infected with *Borrelia* species [14, 15]*. I. ricinus* habitat is expanding to higher latitudes and tick populations are becoming more abundant in established regions [9, 10, 13]. This is likely due to milder winters and a longer vegetation period [8, 9, 12]. The vegetation period is the part of the year in days where the average daily temperature exceeds a certain limit, commonly 5 °C. Ticks are common in the areas of Sweden where the vegetation period is > 180 days. [9, 16]. The vegetation period is increasing throughout Sweden and is projected to keep increasing during this century [11]. This will lead to a longer active period for ticks, which in turn leads to a higher risk of human infection. The climate’s effect on the ticks has been shown through measures of tick populations, but also through incidence of tick-borne disease [8, 11, 13].

Due to the factors mentioned above, the incidence of LB and supposedly LNB is likely to have increased, and to continue increasing. However, as LNB surveillance is lacking in Sweden, the knowledge of LNB epidemiology is limited.

The purpose of this study was to describe the epidemiology of LNB in Kalmar County, Sweden, an area known to be highly LB endemic, between 2008 and 2019, and to investigate the relationship between the LNB incidence and the climate factors precipitation, temperature, humidity, and vegetation period.

## Materials and methods

### Analysis of laboratory data

Two different data sets were received from the departments of clinical chemistry and microbiology at the Kalmar County hospital. These files included raw data with all CSF cell counts and borrelia CSF/serum antibody index results, respectively, from 2008 to 2019 that have been analysed in these laboratories, which cover all of Kalmar County. The data sets were matched to each other by personal identification number and sampling time and date. The data was then anonymised giving each unique individual a code number.

The number of cases in the respective groups below were found monthly and annually. Age and sex distributions were also extracted. Patient data records were not covered and included in the study, thus clinical presentation could not be determined. Only patients with complete CSF/serum pairs originally sampled and sent for borrelia CSF/serum antibody index together with CSF leukocyte counts were included in the study. As these sample pairs originally were sent for LNB diagnostics either to rule in or out LNB diagnosis, these patients were assumed having LNB compatible symptoms and/or clinical findings.

Thus, the following definitions were chosen in this study based on laboratory findings only:

- Definitive LNB refers to cases with positive borrelia antibody CSF/serum index IgM and/or IgG and CSF leukocytes > 5 × 10^6^/L.
- Possible LNB with positive antibody index will refer to cases with a positive borrelia antibody CSF/serum index, for IgG and/or IgM antibodies, but CSF leukocytes ≤ 5 X 10^6^/L.
- Possible LNB with pleocytosis will refer to cases with CSF leukocytes > 5 X 10^6^/L, and a negative borrelia antibody CSF/serum index for both IgG and IgM antibodies.
- Non-LNB refers to cases with a negative borrelia antibody CSF/serum index IgM and IgG and CSF leukocytes ≤ 5 × 10^6^/L.

A detailed log was kept of each step as to easily replicate certain steps and identify errors. Controls were done consistently by choosing random data points in the current working file and comparing to the original, as to check that each test variable stayed matched to the correct personal identification number. Data which did not have a corresponding test to be paired with was excluded, e.g. CSF cell counts without matching borrelia antibody CSF/serum index results. CSF cell counts are more widely used in various medical investigations for other neurological syndromes, including the infectious conditions encephalitis and meningitis, as well as other inflammatory conditions in the CNS. Tests without a unique personal ID number, i.e. patients without Swedish residency, were excluded as the format varied in the files and an accurate match could not be guaranteed.

### Climate data

Total monthly precipitation, mean monthly air temperature, mean monthly relative humidity, and vegetation period were chosen due to previous studies on climate factors, tick activity, and LB [8, 9, 17].

**Fig. 1:**
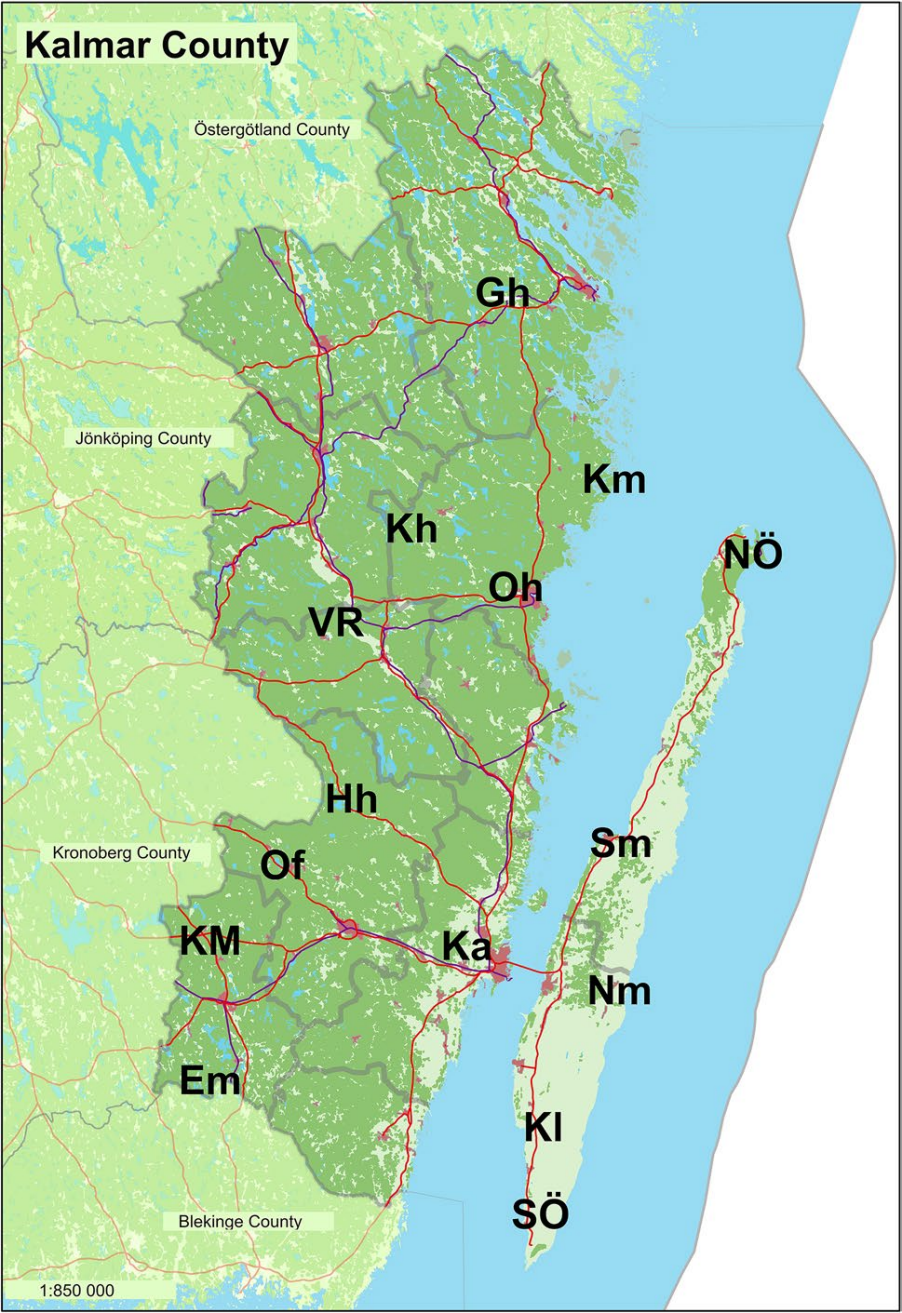
Weather stations in Kalmar County where climate data was collected via the Swedish Meteorological and Hydrologocial Institute. (^a^) indicates areas where mean monthly and hourly temperature was collected, (^b^) monthly precipitation, and (^c^) hourly relative humidity. (Gh) Gladhammar^abc^, (Km) Kråkemåla^b^, (NÖ) northern Öland^abc^, (Kh) Krokshult D^b^, (Oh) Oskarshamn^ab^, (VR) Viserum-Rödmossa^b^, (HH) Hinshult^b^, (Sm) Skedemosse D^b^, (Of) Orrefors^b^, (KM) Kosta Mo^a^, (Ka) Kalmar airport^ab^, (NM) Norra Möckleby D^b^, (EM) Emmaboda D^b^, (Kl) Kastlösa^b^, (SÖ) southern Öland^abc^. Modified map acquired from Lantmäteriet authority in Sweden

Data was retrieved from the Swedish Meteorological and Hydrological Institute (SMHI) website for monthly precipitation, mean monthly and hourly air temperature, and hourly relative humidity [18]. Data was downloaded from stations throughout Kalmar County which were actively collecting the respective data between 2008 and 2019, see Fig. 1, then averaged between stations for each month.

The vegetation period for each year was also calculated. This was accomplished by downloading mean hourly air temperature data from SMHI, from the same six stations as mean monthly air temperature. The number of days counting from the first day of the first 4-day period with a daily mean air temperature > 5 °C until the last day of the last 4-day period with daily mean temperatures > 5 °C were found for each year and station, then averaged for each station and year as to get one vegetation period per year averaged out throughout Kalmar County.

### Statistical analysis

Epidemiological data is presented with descriptive statistics including numbers and percentages. The Pearson chi-square test was used to compare the years with the highest and lowest incidence.

The Spearman’s rank order correlation coefficient was used to examine the association between vegetation period and incidence of LNB (per 100,000 and year). The associations between the other climate factors and incidence of LNB were examined by a series of ordinary least square (OLS) regression analyses. In a first step, the incidence of LNB (per 100,000 and calendar month) was regressed on mean temperature (°C), precipitation (mm), and humidity (%) respectively, using simple OLS regression analyses. In a second step, the incidence of LNB was regressed on all three climate factors using a multiple OLS regression analysis. This multiple OLS regression was conducted to examine the independent association between each climate factor and the incidence of LNB. Further, the regression analyses were conducted with the climate factors of each month matched to the incidence of LNB one and two calendar months after, as to account for the time between tick bite, development of symptoms and the diagnostic lumbar puncture.

The diagnostics of the OLS regression models showed that the residuals were not normally distributed; evaluated with standardized normal probability plot, histogram and the Shapiro–Wilk test. In addition, the assumption of homoscedasticity was violated according to the Breusch-Pagan/Cook-Weisberg test. To handle these problems, both the univariate and multiple OLS regression models were re-analysed with robust standard errors. These sensitivity analyses resulted in the same conclusions and are therefore not further reported. No problems with multicollinearity (variance inflation factor > 2) were identified.

The level of statistical significance was set at p < 0.05. The regression analyses were conducted in Stata 17.0 (StataCorp LLC, College Station, TX, USA). All other analyses were conducted in Microsoft Excel version 2010.

## Results

### General and laboratory results

During the study period, a total of 5318 borrelia CSF/serum antibody index results were identified as well as 8145 CSF leukocyte counts. After matching of data, a total of 5051 CSF/serum pairs with complete data including borrelia CSF/serum antibody index results together with CSF leukocyte counts from 4834 unique patients were identified. Incomplete results, as well as non-human results and failed test results, were excluded from further analysis, see supplementary Table 1.

A total of 205 patients were tested more than once, 11 of these patients were tested three times and one patient four times. The remaining 193 were tested twice.

The population in Kalmar County ranged from 233,090 inhabitants to 244,670 inhabitants during the study period [19].

### Lyme neuroborreliosis in Kalmar County

**Fig. 2:**
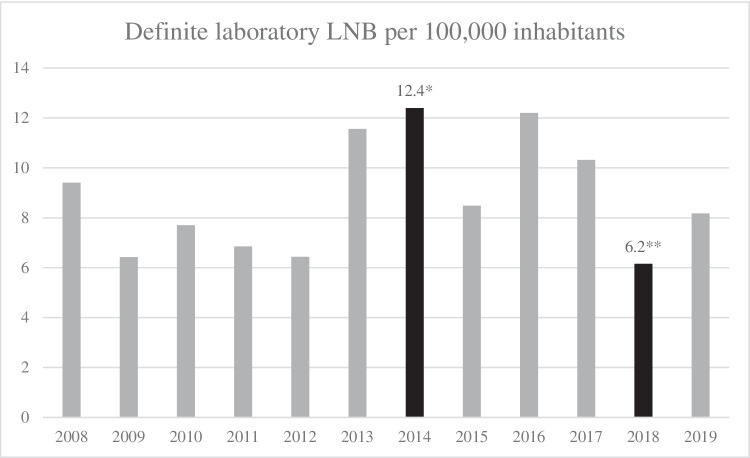
Cases of definite laboratory Lyme neuroborreliosis (LNB) per 100,000 inhabitants in Kalmar County, during the years 2008–2019. *The maximum incidence during the study, 12.4, occurred in 2014, and the **minimum incidence, 6.2, in 2018 (p = 0.025)

**Fig. 3:**
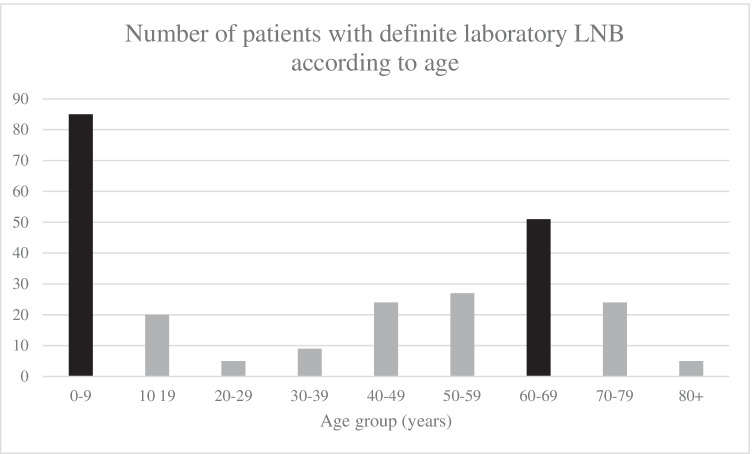
Ages of patients with definite laboratory Lyme neuroborreliosis (LNB) in Kalmar County, during the years 2008–2019. The most cases were found in the age group 0–9 years, followed by 60–69 years

Distribution of the 5051 CSF/serum sample pairs according to diagnostic LNB group, sex and age is shown in Table 1. A total of 251 definitive LNB cases were identified. Definitive cases ranged from a minimum of 6.2 cases per 100,000 inhabitants in 2018, to a maximum of 12.4 cases per 100,000 inhabitants in 2014 (p = 0.025), Fig. 2. The average incidence during 2008–2019 was 8.8 cases per 100,000 inhabitants. The distribution of definite LNB cases according to age groups is shown in Fig. 3 depicting a bimodal distribution pattern with the most cases found in 0–9 and 60–69-year-olds.

**Table 1:** Distribution of identified cases according to Lyme neuroborreliosis diagnostic groups in relation to sex and age

	Total	Definite laboratory Lyme neuroborreliosis; positive antibody index^a^ and pleocytosis	Possible Lyme neuroborreliosis; positive antibody index only	Possible Lyme neuroborreliosis; pleocytosis only	Non-Lyme neuroborreliosis; negative antibody index, no pleocytosis
n	5051	251	88	756	3956
Sex, n (%)					
Female	2612 (51.7)	125 (49.8)	34 (38.6)	360 (47.6)	2093 (52.9)
Male	2439 (48.3)	126 (50.2)	54 (61.4)	396 (52.4)	1863 (47.1)
Age (years)					
Median (IQR) [range]	52 (35) [0–94]	43 (56) [1–91]	61 (22) [5–88]	39 (44) [1–88]	54 (27) [0–94]

n, numbers; ^a^ Positive cerebrospinal fluid – serum anti-*Borrelia* IgM/IgG antibody index; Pleocytosis defined as cerebrospinal leukocytes > 5 * 10^6^/L; IQR, interquartile range

### Climate in Kalmar County

Yearly results on climate factors for Kalmar County during the study period are presented in Table 2. The warmest months were July and August, whereas the months with most precipitation were July and November. The relative humidity was highest in January (87%), and the lowest in August (72%). Higher mean temperature was significantly associated with higher precipitation (r = 0.23, p = 0.005) while none of these climate factors were significantly associated with humidity, r =  − 0.14 and r = 0.09 respectively.

**Table 2:** Climate factors in Kalmar County between 2008 and 2019, collected from the Swedish Meteorological and Hydrological Institute

	Mean (SD) 2008–2019	Maximum (year)	Minimum (year)
Yearly temperature (°C)	7.9 (0.8)	8.8 (2014)	6.03 (2010)
Yearly precipitation (mm)	47.0 (6.3)	62.4 (2010)	38.5 (2018)
Yearly relative humidity (%)	78.4 (8.3)	92.8 (2008)	63.5 (2018)
Vegetation period (days)	245.1 (24.7)	285.2 (2019)	209.2 (2010)

### Associations between climate factors and incidence of Lyme neuroborreliosis

The univariate OLS regression analyses with incidence of LNB one calendar month after registered climate data as outcome variable showed that increased mean temperature and precipitation were significantly associated with increased incidence (p < 0.001, p = 0.003). In contrast, no significant association was shown for humidity (p = 0.928). These associations retained in the multiple OLS regression analysis; the climate factors explained 12% of the variance in incidence. When analysing the incidence of LNB with a delay of two calendar months after noted climate factor data as outcome variable, no significant associations were shown for any of the climate factors in the univariate or multiple OLS regression analyses (Table 3).

**Table 3:** Associations between climate factors and incidence in Lyme neuroborreliosis (per 100,000 and month) based on ordinary least square (OLS) regression analyses

		Univariate OLS regression models			Multiple OLS regression models		
Incidence LNB	Climate factors	B	95% CI for B	p-value	B	95% CI for B	p-value
One month after	Mean temperature	0.036	0.017/0.056	< 0.001	0.031	0.011/0.052	0.003
	Precipitation	0.008	0.003/0.012	0.003	0.006	0.001/0.010	0.022
	Humidity	0.001	− 0.007/0.008	0.928	0.001	− 0.006/0.008	0.761
	Model statistics:				F(3, 139) = 6.38, p < 0.001, R^2^ = 0.121		
Two months after	Mean temperature	− 0.007	− 0.028/0.014	0.530	− 0.001	− 0.033/0.010	0.298
	Precipitation	0.004	− 0.001/0.009	0.116	0.005	− 0.001/0.001	0.077
	Humidity	0.001	− 0.007/0.008	0.957	− 0.001	− 0.008/0.007	0.799
	Model statistics:				F(3, 138) = 1.19, p = 0.315, R^2^ = 0.025		

All findings have been controlled with robust standard errors since the OLS regression models have demonstrated problems with heteroscedacity and/or non-normal distribution of residuals; the results were confirmed for all models in the table but has not been included

The vegetation period was not significantly associated with incidence of LNB (r_s_ = 0.26, p = 0.417).

## Discussion

This study found a generally high LNB incidence of 8.8 cases annually per 100,000 inhabitants, although no overall increasing or decreasing trend was shown in Kalmar County between 2008 and 2019. Interesting relationships between both mean temperature and precipitation and LNB incidence one calendar month later were detected, but no relationship to relative humidity could be established.

### Lyme neuroborreliosis in Kalmar County

Although no overall change in incidence of LNB during the study period could be shown, the annual incidence for the year with highest incidence of LNB (2014) showed a statistically significant difference from the year with the lowest incidence (2018). Notably, the incidence maximum of 12.4 cases per 100,000 inhabitants was observed in 2014, and the incidence minimum, 6.2 cases per 100,000 inhabitants, was observed in 2018. This coincided with the warmest year, 2014, and the driest year, 2018.

The average annual incidence over the study period was 8.8 cases per 100,000 inhabitants, which is higher than the Swedish national incidence in 2014, 6.3 cases per 100,000 inhabitants. The above-mentioned study from 2014 also found the highest annual incidence of LNB along the west and east coast of southern Sweden, with Kalmar County being one of the counties with the highest incidence [6]. This supports our finding of a higher incidence in Kalmar County than the Swedish national average, and also almost twice the mean incidence of LNB in Denmark 1995–2014 of 4.7 per inhabitants per year [20]. This finding also highlights the fact that LNB incidence is the most common bacterial cause of meningitis in Sweden. In comparison, the incidence of other bacterial causes of meningitis taken together in Sweden is given as 2 per 100,000 adult inhabitants per year by the Swedish Infectious Diseases Association [21].

A study from Jönköping County in southern Sweden reported a doubling of LNB incidence from 2000 to 2009 [5]. A Norwegian study also described an increase in LNB incidence in children between 1997 and 2009 [22]. This increase in incidence during the early 2000s is also reported in studies from Denmark and Germany; however, the LNB incidence in these countries then decreased from the late 2000s to the late 2010s. It is not known why the LNB incidence has decreased in these countries. Over the same period in Germany, the total LB incidence, dominated by EM, was unchanged, though LNB incidence decreased [7, 23–26]. It is difficult to compare these trends between countries, due to differing geographical factors, abundance of ticks, and awareness of LB [7]. Awareness of ticks and use of preventive practices are well established in southern Sweden, and this could potentially also affect the incidence [27]. LNB incidence can be affected by many other causes, aside from temperature and preventative practices, such as how much time an individual spends in an area with ticks, as well as when, or if, they seek healthcare and receive a proper diagnosis [28].

LNB cases peaked in July and August and in the age groups 0–9 years and 60–69 years. This is in accordance with previous studies [2, 7, 23, 26]. January through April held the lowest amount of monthly LNB cases, with four cases discovered for each month over the study period. This is probably partially due to tick activity decreasing during the colder months [29]. However, cases are detected all year round, and as there is an individual variation in time from tick bite to neurological symptoms and diagnosis, LNB should still be considered as a possible diagnosis during all months of the year [30, 31]. Our study found no dominance of either sex in LNB cases. Of all patients tested, 48% were male and 52% female. Some studies have reported a slight (55%) dominance of LNB cases in males; however, this was not verified in our study [23–25].

### Lyme neuroborreliosis and the climate

We found positive relationships between both mean temperature as well as precipitation and the incidence of LNB the following calendar month, which is the first time such a relationship has been described to our knowledge. However, no associations were found for the studied climate factors and LNB incidence two calendar months later.

The reason for analysing incidence matched to the climate factors the previous calendar month, as well as two months before, is to compensate for the delay in time from tick bite to onset of neurological symptoms and subsequent medical investigation including lumbar puncture [30, 31]. The length of this delay is hard to study as the causing tick bite many times is difficult to determine. There may also be an individual variation in time for these events to take place, which complicates the analysis. This is exemplified in the fact that cases were found all year around, despite the unlikelihood of a person being bitten by a tick in the winter months. It is, however, unlikely that a patient received a tick bite, started showing neurological symptoms, and had a lumbar puncture done for diagnosis, all within the same month. Therefore, analysis of incidence matched to the climate factors of the same calendar month was not included.

The relationship between temperature and incidence is likely due to ticks being more active and abundant in warmer temperatures, but also due to human behaviour; more likely engaging in various outdoor activities coupled with higher risk of tick exposure, as well as wearing lighter clothing during warmer weather. A positive relationship between temperature and tick activity, as well as EM incidence, have previously been observed, which further supports our findings [8, 9, 13]. Interestingly, and somewhat in contradiction to the findings of Bennet et al. for EM, we also show a positive relationship between precipitation and incidence of LNB the following month. The explanation for this discrepancy may be that we have investigated the LNB incidence the following calendar month, whereas Bennet et al. investigated the mean incidence of EM in the same month.

Our finding, regarding warmer temperatures and increased precipitation correlating to a higher LNB incidence, suggests that health care professionals, as well as the general public, should have increased awareness of LNB symptoms in relation to these weather conditions.

We chose to analyse mean temperature, precipitation and relative humidity based on a previous study in which relationships between these climate factors and EM were found [8]. Although, we in our study show associations between mean temperature as well as precipitation and LNB incidence, we could not establish an association with relative humidity. These differences in relationships between EM and LNB and climate factors are intriguing. Possibly, there were too few cases of LNB in our study to accurately analyse the effect of all climate factors. Interestingly, however, the year with the lowest LNB incidence, 2018, was a year of extreme drought in Kalmar County. It has been observed that ticks are vulnerable to drought and require a certain level of humidity to be able to seek hosts [32]. There may also be large delays in diagnosis which may skew our results. EM is both more common and has a shorter time between tick bite and symptoms, which could make it easier to find these associations.

## Study limitations

Weaknesses in this study include not having access to clinical data, which is required to receive an LNB diagnosis; therefore we have used the term laboratory LNB, which may not be as accurate. However, a study from the Public Health Agency of Sweden found that laboratory data gives a reliable number of cases and is useful for surveillance [6]. Furthermore, the data analysed in our study is collected from standard clinical practice in which only lumbar puncture is performed when symptoms and clinical findings indicate a neurological condition. Moreover, as CSF and serum samples originally were sent for LNB laboratory analyses either to rule in or out LNB, clinical symptoms compatible with LNB in this study and identified patients are assumed and also confirmed by a similar patient cohort in the same geographical area [33].

As mentioned above, the time from tick bite to onset of neurological symptoms varies. In this study, we assumed that a patient received a tick bite one or two months before a diagnostic LP was performed. It is likely that some LNB cases were therefore not accurately matched to the climate factors. However, as stated previously, many patients who receive the LNB diagnosis do not recall a tick bite, so clinical data would have limited helpfulness.

## Conclusions

A high LNB annual mean incidence of 8.8 cases per 100,000 inhabitants was found in Kalmar County, but no overall change in incidence trend could be detected in 2008–2019. The incidence peaked in July and August as well as within age groups 0–9 years and 60–69 years, in accordance with previous studies. Cases were diagnosed in all months of the year, suggesting that clinicians should not discount LNB as a differential diagnosis during the winter or spring months.

We also conclude positive relationships between mean temperature as well as precipitation and LNB incidence the following calendar month. This may be due to tick abundance and activity, as well as human outdoor activity, resulting in changes in tick-human interactions, and suggests increased awareness of LNB under these circumstances as well as in relation to possible climate change. We found no relationship between relative humidity, or vegetation period and LNB incidence.

The reasons for these relationships, and non-relationships, have not been established. Further studies with larger study groups, covering other geographical areas and over longer time are needed to understand these findings. Further, such studies, as well as general monitoring of the development of LNB, would be facilitated by making LNB a notifiable condition across Europe including Sweden.

## Supplementary Information

Below is the link to the electronic supplementary material.

Supplementary file1 (DOCX 17 KB)

## Data Availability

Anonymized data is available from the corresponding author upon reasonable request.

## Code availability

Code is available upon request.
