# Supplementary material for: The relationship between the laboratory diagnosis of Lyme neuroborreliosis and climate factors in Kalmar County Sweden — an overview between 2008 and 2019
Source: Eur J Clin Microbiol Infect Dis. 2021 Nov 9;41(2):253–61. doi: 10.1007/s10096-021-04374-4 (PMC8770396; doi:10.1007/s10096-021-04374-4)
Supplement: Supplementary file 1 — Supplementary file1 (DOCX 17 KB) [file 10096_2021_4374_MOESM1_ESM.docx]

| Supplementary Table 1. Information on excluded data | | | | | | |  | |
| --- | --- | --- | --- | --- | --- | --- | --- | --- |
| Year | Total | Antibody index^a^ result, but no matching CSF leukocyte count | CSF leukocyte count, but no matching antibody index result | No Swedish identification number (experimental- dogs/external controls) | Other^b^ |  | |  |
| 2008 | 279 | 22 | 255 | 2 | 3 |  | |  |
| 2009 | 279 | 23 | 252 | 2 (2) | 8 |  | |  |
| 2010 | 299 | 19 | 275 | 5 | 7 |  | |  |
| 2011 | 270 | 25 | 245 | 0 | 7 |  | |  |
| 2012 | 293 | 25 | 266 | 2 | 8 |  | |  |
| 2013 | 270 | 27 | 240 | 3 | 3 |  | |  |
| 2014 | 318 | 18 | 296 | 4 | 9 |  | |  |
| 2015 | 273 | 18 | 245 | 6 (4) | 1 |  | |  |
| 2016 | 318 | 36 | 270 | 9 (3) | 2 |  | |  |
| 2017 | 276 | 17 | 256 | 3 | 5 |  | |  |
| 2018 | 237 | 20 | 216 | 1 | 5 |  | |  |
| 2019 | 264 | 15 | 246 | 3 | 2 |  | |  |
| Total | 3436 | 265^c^ | 3062 | 49 | 60 |  | |  |
| CSF, Cerebrospinal fluid  ^a^ Cerebrospinal fluid – serum anti-*Borrelia* IgM/IgG antibody index  ^b^ Other includes tests where a result was not given for reasons including coagulation, deleted result, or a reference to a comment, which we did not have access to.  ^c^ Out of these 265 antibody index results with no matching cell count, were 15 positive antibody index results | | | | | | | . | |
